# Mitigating Long-Term COVID-19 Consequences on Brain Health

**DOI:** 10.3389/fneur.2021.630986

**Published:** 2021-09-27

**Authors:** Ryan C. N. D'Arcy, Jagdeep K. Sandhu, Shawn Marshall, Markus Besemann

**Affiliations:** ^1^Centre for Neurology Studies, HealthTech Connex, Vancouver, BC, Canada; ^2^Faculty of Applied Sciences, Simon Fraser University, Vancouver, BC, Canada; ^3^DM Centre for Brain Health (Radiology), University of British Columbia, Vancouver, BC, Canada; ^4^Human Health Therapeutics Research Centre, National Research Council Canada, Ottawa, ON, Canada; ^5^Department of Biochemistry, Microbiology and Immunology, University of Ottawa, Ottawa, ON, Canada; ^6^Physical Medicine and Rehabilitation, University of Ottawa, Ottawa, ON, Canada; ^7^Rehabilitation Medicine, Canadian Forces Health Services, Ottawa, ON, Canada

**Keywords:** SARS-CoV-2, coronavirus, brain inflammation, cognition, neuromodulation, neuroplasticity, mental health, moral injury

## Abstract

COVID-19 is increasingly being linked to brain health impacts. The emerging situation is consistent with evidence of immunological injury to the brain, which has been described as a resulting “brain fog.” The situation need not be medicalized but rather clinically managed in terms of improving resilience for an over-stressed nervous system. Pre-existing comparisons include managing post-concussion syndromes and/or brain fog. The objective evaluation of changes in cognitive functioning will be an important clinical starting point, which is being accelerated through pandemic digital health innovations. Pre-morbid brain health can significantly optimize risk factors and existing clinical frameworks provide useful guidance in managing over-stressed COVID-19 nervous systems.

## Introduction

The Coronavirus 2019 (COVID-19) pandemic has manifested in many clinical presentations. Central nervous system (CNS) involvement is not a rare complication of this virus. In the current analysis, we postulate that: (1) pre-morbid brain health may be a significant modifiable risk factor when considering clinical sequelae; and (2) a framework similar to concussion management may provide helpful guidance in next-steps for COVID-19 clinical management. In this way, CNS related concerns can be represented as treating an “over-stressed” nervous system. It is not desirable, from a public health perspective, to create a unifying diagnosis that potentially “medicalizes” COVID-19 related CNS symptomatology and dysfunction to the detriment of empowering environmental and lifestyle choices that are within a patient's control.

This article first summarizes current state of knowledge pertaining to neuroinflammation and the neurological consequences related to COVID-19, then suggests “brain health” risk factors amenable to modification, and proposes means to measure cognitive brain function in a cost-effective and efficient manner, with simple strategies to potentially mitigate long-term consequences of post COVID-19 “brain fog.” Similar concepts around brain fog have been identified in association with concussion, anoxic insult, and chemotherapy. While any one of these may serve as a potential model, given its prevalence the concussion model is adopted in this brief analysis.

## Immunological Injury to the Brain

CNS involvement with COVID-19 is real, measurable, and potentially modifiable. Increasing evidence has demonstrated that COVID-19 is associated with CNS dysfunction, as demonstrated by a range of neurological and mental health symptoms spanning from acute to potentially chronic conditions (see literature review below). We propose an approach to prevent, mitigate and rapidly identify the small but significant minority of those with residual symptoms realizing that the reasons for residual symptoms might be complex and multiple. The underlying pathophysiology of this involvement appears to revolve around neuroinflammation, a common factor amongst many conditions affecting neurological function. The propensity toward inflammation is dependent on many variables, of which some are modifiable. Consequently, there is a constellation of physical, psychological, and cognitive factors that contribute to whether or not a specific injury leads to functional disability or not. This model is not dissimilar to those currently in place for concussion care (1).

SARS-CoV-2 was initially thought to infect primarily the lower respiratory tract and cause lung damage. However, a growing body of evidence suggests that under certain circumstances SARS-CoV-2 can infect the CNS and cause neurological complications (2). It was clear even in the early months of the COVID-19 pandemic that neurological manifestations, such as headache, dizziness, confusion, ageusia and anosmia were common in more than 50% of the hospitalized COVID-19 patients (3, 4). Surprisingly, acute cerebrovascular disease with increased risk of stroke is also emerging as an important neurological complication in hospitalized patients with severe COVID-19 disease (5, 6). The long-term consequences of these symptoms on brain health may not be realized for years or decades. While our knowledge on the neurological impacts of SARS-CoV-2 continues to evolve, the lessons learnt from recent studies can provide an important roadmap to advance our understanding of SARS-CoV-2 pathogenesis (7–9). Neuropathological examination of post-mortem brain specimens obtained from COVID-19 patients have shown acute hypoxic-ischemic changes in the cerebellum and loss of neurons in cerebral cortex, hippocampus and cerebellar Purkinje cell layer. Further testing of brain tissue using molecular and immunohistochemical analysis revealed the presence of low levels of viral ribonucleic acid (RNA) or nucleocapsid proteins (9). In a case series study, early post-mortem brain magnetic resonance imaging (MRI) scans of COVID-19 patients demonstrated hemorrhage, posterior reversible encephalopathy syndrome and non-specific deep white matter changes, possibly due to the blood-brain barrier (BBB) breakdown (8). Another study showed elevated plasma levels of neurofilament light chain protein (NfL), a marker of neuronal injury and glial fibrillary acidic protein (GFAP), a marker of astroglial injury in COVID-19 patients, suggestive of direct CNS damage (7). Although these studies provide evidence of CNS damage in COVID-19 patients, whether these lesions are due to direct viral infection of the brain could not be established and further mechanistic studies are warranted.

The blood-brain barrier (BBB) represents a formidable barrier that prevents harmful substances, including viruses from entering the CNS. SARS-CoV-2 possibly deploys several strategies to evade the innate immune responses, traverse the BBB and gain entry into the CNS (10). The spike protein of SARS-CoV-2 binds the angiotensin converting enzyme-2 (ACE-2) receptor to infect brain endothelial cells and activate inflammatory and thrombotic pathways (11, 12). Given the importance of the BBB for the maintenance of cerebral blood flow, cerebrovascular endothelial cell dysfunction could lead to alterations in BBB function. SARS-CoV-2 infection has been associated with meningitis and pan-encephalitis and viral RNA was detected in the cerebrospinal fluid (CSF) of a COVID-19 patient (13, 14). A case of acute hemorrhagic necrotizing encephalitis on MRI scans has been reported in COVID-19 patients, suggestive of hyperinflammation (15). In addition, increased serum and CSF levels of proinflammatory cytokines, IL-1β, and IL-6 have been reported in SARS-CoV-2-associated encephalitis (16). Systemic exposure to pathogenic levels of proinflammatory mediators could result in immunopathogenic and neuronal injury (17), eventually leading to cognitive dysfunction. Similar to the 1918 Spanish Flu pandemic, with reports of encephalitis lethargica and post-encephalitis parkinsonism (18), case study evidence of parkinsonism has emerged after SARS-CoV2 infection (19). Therefore, it is important to diagnose and treat the neurological manifestations in COVID-19 patients at an early stage in the disease process to limit the long-term sequelae.

## Factors Affecting Resilience and Recovery

Public health measures to limit the transmission of SARS-CoV-2 have also consequently impacted conventional evaluation and treatment options and practices. Like any complex condition, a bio-psycho-social model is needed that integrates the inflammation effects with those related to psychosocial and other stressors. CNS care in a COVID-19 era involves treating an already over-stressed nervous system. Care can involve addressing multiple factors spanning from direct neurological injury, mental health, post-traumatic stress disorder (PTSD), and potentially extending to the emerging concept of “moral injury” when the impact to front-line responders and many others is also considered.

In this respect, COVID-19 can expose vulnerabilities in brain resiliency and challenge a host of underlying factors that support brain health. Cognitive and mental health complaints appear to be emerging as early reported symptoms (20). In essence, COVID-19 has stressed the nervous system, but a system that in many cases was already stressed to begin with. As any biological homeostatic system, resilience can be seen as a common thread that brings together CNS injury, cognitive functioning, general mental health and resiliency, PTSD, and moral injury. Prevention and recovery therefore involves searching for links (diet, exercise, sleep, et cetera) to some key factors that can be influenced in order to manage common symptoms that have previously not been understood to be connected.

## Clinical Management Next Steps

By comparison, all consensus guidelines for concussion management show that reassurance and expectation for a positive outcome are the single most important factors in management. It is reasonable to assume that for a multi-factorial condition such as COVID-19, barring any evidence to the contrary, a similar approach should be implemented. Given the significant amount of media-coverage and the already escalating toll on mental health, public policies that encourage and promote pro-active approaches to pre-habilitation and “normalization” of non-specific symptoms might avoid potentially inappropriate and costly labeling of non-specific neurological symptoms. As any practicing neurologist will certainly attest, there is no shortage of “functional neurological disorder” in daily practice. A consensus statement has been recently published to guide rehabilitation post-COVID-19 (21).

A recent review has outlined five major categories of modifiable personal factors that contribute to baseline brain health and which may be protective against various CNS conditions: (1) exercise; (2) cognitive stimulation; (3) sleep; (4) dietary considerations; and (5) social connectedness (22). The detailed review of these is beyond the scope of this article, but a few points are worthy of mention. The effects of physical activity on brain function are well-established. The effects of brain-derived neurotrophic factor (BDNF) on neuroplasticity and neuromodulation are well-known. Regrettably, with some exceptions, COVID-19 public health measures have affected the access to exercise facilities or outdoor activity for many. Physical activity needs to increase prominently in public health messaging as lack of exercise is a major cause of chronic diseases (23). In the now famous video “23 ½ h,” Dr. Michael Evans makes a very compelling argument that it only takes 30 min of brisk walking per day to achieve most exercise related health benefits (24). Clearly this mode of exercise should be available to all in a COVID-19-safe manner. The role of cognitive stimulation has gained greater awareness, with various applications and technologies available to help facilitate cognitive and mental well-being (25). The importance of sleep and diet for brain health has been validated and amply documented, with dietary considerations particularly focused on anti-inflammatory diets (e.g., omega-3 fatty acids and medium chain triglyceride supplementation, magnesium, Intermittent fasting and Ketosis, etc.) (26). Simple modifications in this domain could have significant impacts in reducing the pre-morbid burden of dietary-related CNS inflammation that may predispose individuals to an “inflammatory storm” that may have otherwise been potentially less severe. Finally, if social connectedness is key to cognitive and brain health, there is an interesting paradox brewing insofar as the precise tools used to contain the spread of disease may be contributing directly or indirectly to long-term disease burden from a CNS perspective.

## The Importance of Cognitive Function

By the same token, measuring cognitive function in an objective manner would allow for serial tracking of CNS involvement and recovery. In contrast to traditional neuropsychological testing, which can be time-consuming and is increasingly challenging to access in a post-COVID-19 era, there have been several recent digital health advances in cognitive evaluation. A number of user-friendly, portable cognitive/behavioral evaluation technologies have been developed (e.g., BrainFX, CBS Health, BrainHQ, etc.). Novel portable neurophysiological options are also emerging. In terms of the most recent advances in neurotechnology capable of deployment to clinical frontlines, portable electroencephalography (EEG) can now provide non-invasive, objective, neurophysiological, monitoring systems (e.g., BrainScope, NeuroCatch® Platform, and eVox System) and may prove beneficial in mitigating the long-term consequences of cognitive impairments from COVID-19. The key clinical management advance here is early and sensitive evaluation, which can lead to earlier and more effective interventions.

As the medical axiom states: You can't treat what you can't measure. In HIV, it took more than a quarter of a century to establish the HIV-associated neurocognitive disorder HAND (27). Accordingly, it will be important to start with evaluating basic cognitive complaints within the framework of an over-stressed nervous system and anticipate the rise of descriptors such as “brain fog” similar to that which occurs in concussion. Historically, rapid, objective, and accurate clinical evaluation of cognitive function has been difficult, particularly now in the pandemic era. However, digital health advances have overcome the inherent challenges of cognitive screening and neuropsychological testing that rely on subjective behavioral responses that can be error prone (28). Through the use of portable EEG, it is possible to obtain objective neurophysiological testing through low-cost, clinically accessible technologies (29). These devices have been used, particularly for point-of-care concussion management, and are actively used in the current pandemic era (30).

Specifically, cognitive evoked potentials are increasingly being shown to be useful within a vital sign framework that overcomes historical clinical limitations of EEG. The timing for such an advance is critical. Recent CNS COVID-19 reports have highlighted the importance of objectively monitoring vital neurocognitive functions over the longer term (30). Modeled from vital sign frameworks, cognitive brain function can be monitored as an objective, sensitive, physiological evaluation of evoked responses that is clinically accessible, particularly in pandemic times (31–35). The basic framework uses a portable EEG for rapid, automated, standardized evaluation of three established cognitive evoked potentials for Auditory sensation (36), Basic attention (37), and Cognitive processing (38) in under 10 min. In a post-pandemic era, the deployable features of this type of digital health technology are becoming increasingly important tools to investigate subjective cognitive complaints. The detection of subtle cognitive changes may provide further alignment between COVID-19 and concussion in terms of mitigation strategies. Here, emerging cognitive rehabilitation advances will be important to begin evaluating in future prospective studies.

## Discussion

COVID-19 has pushed our personal and collective resources to their limits, with evidence-based impacts on cognitive and related brain health issues. The good news remains that there is much that can be done to improve individual resilience to CNS injury/inflammation.

As we have seen with the relative concussion epidemic, in order to fully understand multisystem, complex issues one needs to start at the beginning with simple yet solid foundational evidence-based interventions that are: (1) Cost effective; (2) Clinically effective; and (3) Result in functional improvements or, at the very least, mitigate functional impairment and (4) Can be implemented on a large-scale.

What other epidemics and pandemics have taught us is that, reducing complex problems to simple organ system injury models rarely provides tangible and lasting solutions at least in the domain of human functioning and participation in life [as per the World Health Assembly's International Classification of Functioning, Disability and Health, (39)]. In fact often, well-intentioned medically focussed interventions can inadvertently contribute to the problem for which the solution was intended. Notwithstanding the immense and commendable work that is being done in the realm of these other conditions, it is paramount that over-medicalization or dramatization of CNS symptomatology and dysfunction be kept to the minimum required to recognize and treat serious pathology but not to create a new pandemic of “worried-well” as has been the case in other areas of health care, specifically in the case of concussion. In the current analysis, we conclude that pre-morbid brain health optimization can significantly modify the risk factors and that a framework similar to concussion management provides useful guidance in clinically managing over-stressed nervous systems.

## Data Availability Statement

The original contributions presented in the study are included in the article/supplementary material, further inquiries can be directed to the corresponding author/s.

## Author Contributions

RD and JS contributed to initial concept and perspectives. RD, JS, and MB developed the initial version of the manuscript. SM reviewed and edited the manuscript along with all other authors. All authors read and approved the final manuscript.

## Funding

This publication and related open access publication fees was supported by HealthTech Connex's Centre for Neurology Studies (RD).

## Conflict of Interest

RD is the inventor of the NeuroCatch Platform medical device and qualifies for financial benefit from commercialization. The remaining authors declare that the research was conducted in the absence of any commercial or financial relationships that could be construed as a potential conflict of interest.

## Publisher's Note

All claims expressed in this article are solely those of the authors and do not necessarily represent those of their affiliated organizations, or those of the publisher, the editors and the reviewers. Any product that may be evaluated in this article, or claim that may be made by its manufacturer, is not guaranteed or endorsed by the publisher.
